# Clinical outcomes and surgical strategy for spine tuberculosis: a systematic review and meta-analysis

**DOI:** 10.1007/s43390-023-00785-9

**Published:** 2023-11-17

**Authors:** Jainal Arifin, Karya Triko Biakto, Muhammad Phetrus Johan, St. Fatimah Zahrah Anwar

**Affiliations:** Department of Orthopaedics and Traumatology, Hasanuddin University, Jl. Perintis Kemerdekaan No. 10, Tamalanrea Indah, Makassar, Sulawesi Selatan 90245 Indonesia

**Keywords:** Tuberculosis, Tuberculous spondylitis, Surgery

## Abstract

**Purpose:**

Spinal tuberculosis (TB) is a slow-developing disease that often causes cord compression, spinal instability, and deformity. Surgery is generally required in cases of refractory disease, severe kyphosis, neurological deficits, or lack of improvement. However, there is a lack of comprehensive evidence in comparing the efficacy of various surgical approaches. The study aims to provide a clearer understanding of the relative effectiveness of the available surgical modalities in the management of spinal TB.

**Methods:**

This review adhered to the PRISMA statement with searching conducted until 11th April 2023. Inclusion criteria included studies involving surgical procedures for spinal tuberculosis, with relevant clinical outcomes reported. Data extraction involved the collection of information on study and population characteristics, interventions used, relevant clinical outcomes, and reported complications. The risk of bias was evaluated using Cochrane’s Risk of Bias in Non-randomized Studies tool.

**Results:**

Searching resulted in 20 cohort studies that analyzed surgical methods for spinal tuberculosis. Eleven studies had low bias and nine studies had moderate bias. The anterior approach was associated with faster perioperative duration [− 2.02 (− 30.71, 26.67), *p* < 0.00001], less blood loss [− 4242 (− 176.02, 91.18), *p* < 0.00001], shorter hospitalization [− 0.19 (− 2.39, 2.01), *p* < 0.00001], better angle correction [1.01 (− 1.82, 3.85), *p* < 0.00001], and better correction rates [11.36 (− 7.32, 30.04), *p* < 0.00001] compared to the posterior approach. Regarding neurological function recovery, the anterior and posterior approaches were equally effective, while the posterior approach was associated with a higher incidence of complications. The review also reported on the complications associated with the surgical approaches, and 9 out of 20 studies reported complications. The anterior approach was found to have fewer complications overall.

**Conclusion:**

The anterior approach is thought to have fewer complications than both combined and posterior-only approaches, but the variability of the findings indicates that the decision-making process for selecting a surgical approach must consider individual patient and disease characteristics, as well as surgeon training.

## Introduction

Tuberculosis (TB) is one of the leading causes of infectious disease-related deaths in developing countries. The prevalence of pulmonary TB in Indonesia in 2018 reached 0.49%, or about 1 million people [1]. The most common site of TB dissemination in bones is in the spine (50%), which accounts for about 1–3% of all TB cases [2, 3]. Patients with spinal TB may also have pulmonary TB in one-third to two-thirds of cases. The most common route of TB dissemination to the spine is hematogenous spread. Spinal TB usually develops slowly, so there is often a period of several months between the onset of symptoms and appropriate medical treatment [2, 4].

Spinal TB often causes cord compression, spinal instability and deformity, requiring surgical treatment [5]. Anterior debridement, bone grafting, and fixation have been used to treat anterior and middle column destruction by TB [6]. The surgical approach can be either from the front (anterior) or from the back (posterior). The surgery may involve only removing damaged tissue (debridement), or a more extensive approach involving complete removal of the affected area followed by autografting (using bone from the patient’s own body) and instrumentation (inserting rods, screws, or plates) [3].

There is still a lack of comprehensive and updated evidence on the comparative efficacy of various surgical approaches used in the treatment of spinal TB. There is still a need for more reliable and efficacious surgical procedures aimed at producing better clinical outcomes in spinal TB patients. Therefore, a systematic review would help fill this knowledge gap by synthesizing the available evidence and providing clinicians with a clear understanding of the relative effectiveness of the available surgical modalities in the management of spinal TB. This study aims to provide clinicians and patients with better decision-making in the clinical setting.

## Methods

### Searching and screening strategy

This systematic review was conducted in accordance to Preferred Reporting Items for Systematic Reviews and Meta-Analysis (PRISMA) statement [7]. Searching was done in PubMed, Cochrane, EMBASE, ProQuest, and SCOPUS, covering all eligible articles up to 11^th^ April 2023. The search process was conducted independently by all authors. Articles were screened using specific keywords such as “tuberculous spondylitis”, “surgery”, and “outcome”. The specific search terms used for each database can be seen in Appendix 1. The initial search results were then deduplicated to find duplicate findings using automation tools (Mendeley) and more search results were removed before screening if they are marked ineligible by the automation tools. The remaining articles were then screened further using the predetermined inclusion and exclusion criteria. The screening process was proceeded with initial screen process based on the abstract with remaining articles screened with full-text screening based on the eligibility criteria.

### Inclusion criteria

Articles on surgery techniques and outcomes on tuberculosis spondylitis or spinal TB were included in this study. The following inclusion criteria were used: (1) interventional or observational studies, (2) patients or participants diagnosed with spinal tuberculosis, (3) intervention being any sort of surgical procedures in managing spinal tuberculosis, (4) comparison done either with a control or another surgical procedure if comparing effectiveness, (5) studies reporting any relevant clinical outcome that may affect or be affected with the surgical procedure.

### Exclusion criteria

Articles with ineligible study design including reviews, comments, case reports, letters, or conference abstracts and studies not in English or Indonesian were excluded. Articles with no comparison of surgical procedure or incomplete nor clinical outcome affected by the surgery were also excluded.

### Data extraction

We extracted the following data from the selected studies: (1) first author’s name and publication year, (2) study characteristics (study design, number of participants, follow-up period), (3) population characteristics (mean age, gender distribution, pathological region, number of involved vertebrae, mean kyphotic angle, disease duration, impairment of neurological function, pre-surgical treatment, and other comorbidities), (4) intervention and control used, (5) relevant clinical outcomes (operation duration, blood loss, correction rate, recovery of neurological function, recovery of neurological function, fusion times, post-operative ESR and CRP, and hospital stay), and (6) complications reported.

### Risk of bias assessment

Risk of bias was evaluated using Cochrane’s Risk of Bias in Non-randomized Studies—of Interventions (ROBINS-I) [8]. Critical appraisal was conducted by two independent authors, which is further adjudicated by a third author if any discrepancies arise. Disagreements were resolved through discussion until common understanding is met. The following were evaluated for bias assessment: (1) bias due to confounding, (2) bias in selection of participants into the study, (3) bias in classification of interventions, (4) bias due to deviations from intended interventions, (5) bias due to missing data, (6) bias in measurement of outcomes, (7) bias in selection of the reported result.

### Statistical analysis

Review Manager software (version 5.3, offered by Cochrane) was used to conduct the meta-analysis. The Chi-square test and I^2^ statistic were used to assess study heterogeneity. A *p* value for the Chi-square test more than 0.1 or an *I*^2^ greater than 50% revealed considerable heterogeneity. A fixed-effect model (Mantel–Haenszel method) was employed for analysis if heterogeneity was negligible; otherwise, a random-effect model was used. *p* values less than 0.05 were considered statistically significant. Sensitivity analysis was carried out using the Duval and Tweedie’s trim-and-fill method until the favorable heterogeneity was met.

## Results

### Study selection

**Fig. 1:**
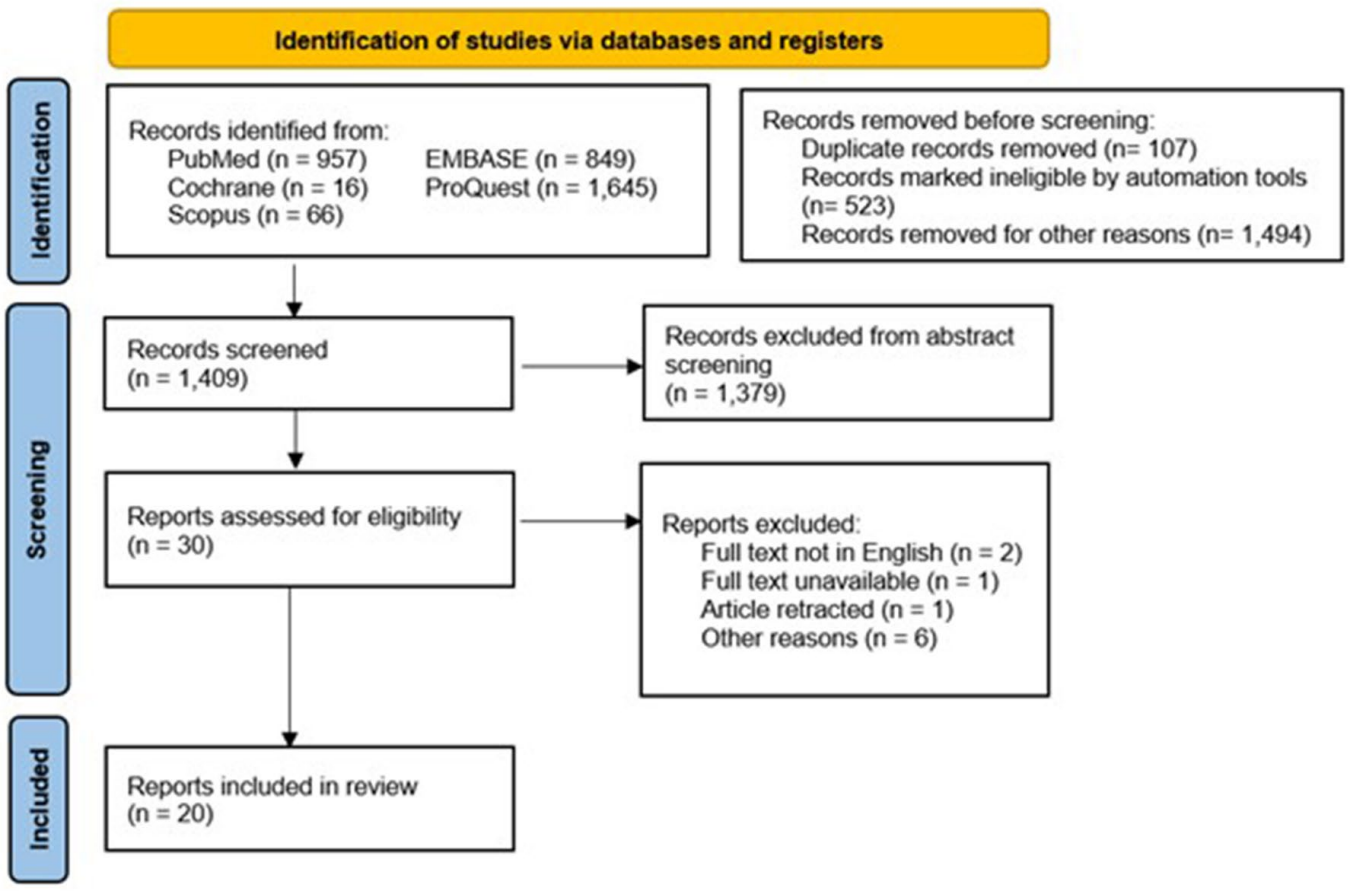
Systematic review article selection process as shown in a Preferred Reporting Item for Systematic Reviews and Meta-Analyses (PRISMA) flow diagram

A total of 1409 were identified for preliminary screening after duplicates were excluded. One thousand three hundred seventy-nine studies were further excluded based on their titles and abstracts alone which yielded thirty studies. Ten studies were then excluded due to not being written in English, unavailable full text, retracted by the journal, or any other reasons. Twenty cohort studies analyzing different surgical methods for spinal tuberculosis were included in this study and further extracted for analysis. Figure 1 shows the study selection process according to the PRISMA flowchart. Data extracted was then summarized into tabular format.

### Study characteristics

Table 1 shows the summarized characteristics of the included studies [9–28]. Accumulatively, 1,415 patients were included in this review. The average age of patients ranges from 6.2 ± 1.1 to 47.5 ± 15 years old. Fifteen studies reported the mean kyphotic angle which ranges from 4.6° ± 17.6 to 74.6°. Six studies reported the disease duration which ranges from 5.3 ± 1.6 to 27.01 ± 7.33 months. Impairment of neurological function was mostly reported using the Frankel scale or the ASIA grade. Only two studies reported the underlying comorbidities present in the included patients. Hypertension, chronic obstructive pulmonary disease (COPD), and diabetes mellitus (DM) were among the most reported comorbidities [9, 25].

**Table 1:** Characteristics of included studies

Authors	Study design	Number of participants	Follow-up period (months)	Population							
				Average age	Gender distribution (M:F)	Pathological region (T/TL/L)	No of involved vertebrae	Mean kyphotic angle	Disease duration	Impairment or neurological function	Other comorbidities
Zhang, 2012 [9]	Retrospective cohort	Group A (intervention): 20 Group B (control): 16	35.1 ± 5.8 months	Group A: 68.6 ± 3.2 Group B: 68.2 ± 3.1	22:14		1: 6 patients 2–3: 25 patients > 3: 5 patients	Group A: 31.9 ± 6.6 Group B: 33.1 ± 7.4		Frankel scale: B: 1 C: 13 D: 11 E: 11	Hypertension (27), COPD (13), diabetes (25), CHD (22), ECG abnormality (19)
Pu, 2012 [10]	Retrospective cohort	Group A (intervention): 25 Group B (control): 22	22.2 (range 12–62) months	Group A: 38.1 Group B: 37.8	17:30	Group A: 7/10/8 Group B: 6/10/6		Group A: 16.3 Group B: 24.4		ASIA grade: Group A: 10 patients in grade D Group B: 13 patients in grade C or D	
Huirong, 2017 [11]	Retrospective cohort	Group A (intervention): 48 Group B (control): 50	26.79 ± 5.43	Group A: 50.05 ± 6.98 Group B: 49.17 ± 7.32	Group A: 28/20 Group B: 28/22				Group A: 27.01 ± 7.33 Group B: 26.38 ± 6.84		
Yuliang, 2017 [12]	Prospective cohort	Group A (intervention): 52 Group B (control): 38		Group A: 42.3 ± 11.5 Group B: 43.8 ± 12.0	Group A: 33/19 Group B: 21/17	T/TL Group A: 7/45 Group B: 20/18				Frankel scale Group A: 0/3/14/19/16 Group B: 0/2/15/12/9	
Li, 2019 [13]	Retrospective cohort	Group A (intervention): 39 Group B (control): 48	6.2 ± 1.1 years	Group A: 35.1 ± 8.4 Group B: 37.2 ± 7.9	Group A: 22/17 Group B: 30/18			Group A: 21.0 ± 5.1 Group B: 22.6 ± 3.0	Group A: 9.6 ± 2.4 Group B: 8.9 ± 2.3		
Sun, 2019 [14]	Retrospective cohort	Group A (Intervention): 18 Group B (control): 14	Group A: 39.1 ± 12.0 Group B: 40.7 ± 12.4 months	Group A: 36 Group B: 44		L/S Group A: 4/14 Group B: 1/13		Group A: 21.04 ± 4.69 Group B: 23.65 ± 1.19			
Demirel, 2019 [15]	Retrospective cohort	Group A (intervention): 16 Group B (control): 15	Group A: 29 months Group B: 28 months	Group A: 56 (29–75) Group B: 60 (35–73)	Group A: 9/7 Group B: 7/8	Group A: 11/3/2 Group B: 10/3/2		Group A: 32.6 ± 14.8 Group B: 25.9 ± 12.7		ASIA scale Group A: 1/1/5/3 Group B: 1/0/2/1	
Zhao, 2020 [16]	Retrospective cohort	Group A (intervention): 19 Group B (control): 16	37.9 ± 11.4	Group A: 35.0 ± 11.8 Group B: 35.0 ± 11.8	Group A: 8/11 Group B: 8/8			Group A: 36.2 ± 15.2 Group B: 27.9 ± 7.7			
Musali, 2020 [17]	Retrospective cohort	30 patients		Group A: 37.8 0 ± 15.36 Group B: 41.8 7 ± 14.14				Group A: 19.4 ± 4.5 Group B: 25.58 ± 7.83			
Qiu, 2022 [18]	Retrospective cohort	Group A (intervention): 24 Group B (control): 28	Group A: 27.2 (13.1–35.3) Group B: 22.3 (16.2–38.0)	Group A: 41.1 ± 19.3 years Group B: 43.2 ± 17.1	Group A: 12/12 Group B: 17/11	Group A: 9/11/8 Group B: 13/10/9	Group A: 2.0 (1.3–2.8) Group B: 2.0 (2.0–2.0)			ASIA grade (C/D/E) Group A: 2/5/17 Group B: 0/10/18	
Jiang, 2022 [19]	Retrospective cohort	Group A (intervention): 36 Group B (control): 31	Group A: 43.33 ± 8.6 Group B: 39.6 ± 10.1	Group A: 71.3 ± 4.5 Group B: 69.9 ± 3.6	Group A: 20/16 Group B: 17/14	Group A: 23/5/8 Group B: 20/3/8		Group A: 26.1 ± 9.1 Group B: 25.5 ± 8.4		ASIA grade (A/B/C/D/E) Group A: 0/4/6/18/8 Group B: 0/3/7/12/9	
Wu, 2022 [20]	Retrospective cohort	Group A (intervention): 40 Group B (control): 47	34.3 ± 9.5 months	Group A: 52.2 ± 13.4 Group B: 50.3 ± 9.9	Group A: 24/16 Group B: 31/16			Group A: 11.2 ± 2.6 Group B: 11.2 ± 2.6	Group A: 5.3 ± 1.6 Group B: 5.3 ± 1.6		
Zhang, 2012 [21]	Retrospective cohort	Group A (intervention): 19 Group B (control): 18	Group A: 46.6 ± 16.7 Group B: 47.5 ± 15.0	41.2 ± 14.7	19:18			Group A: 23.9 ± 7.6 Group B: 28.5 ± 6.5		Frankel scale: B: 6 C: 8 D: 19	
Garg, 2012 [22]	Retrospective cohort	Group A (intervention): 34 Group B (control): 36		Group A: 34.9 (21–50) Group B: 33.6 (18–56)	52/18	T/TL Group A: 16/18 Group B: 20/16		Group A: 44.6° (25°–58°) Group B: 74.6° (48°–86°)	Group A: 10.2 (5–14) Group B: 9.7 (6–13)	Frankel score (A/B/C/D/E) Group A: 0/0/18/12/4 Group B: 0/0/19/11/6	
Wang, 2014 [23]	Prospective cohort	115 patients; Group A (intervention): 55 patients; Group B (control): 60 patients		48.6 ± 12.8	68/47	Group A: 15/28/12 Group B: 18/31/11				ASI grade (Group A/B): A: 0/0 B: 4/2 C: 19/17 D: 21/26 E: 11/15	
Shi, 2014 [24]	Retrospective cohort	148 patients; Group A (nails + screws): 78; Group B (pedicle screws): 48; Group C (arch fixation): 16; Group D (posterior arch fixation): 6	17.9 ± 4.9	39.7 ± 12.3	92/56	Group A: 33/31/14 Group B: 8/16/24 Group C: 0/4/12 Group D: 2/4/0	1: 83 2: 88 3: 54 4: 16		7.3 ± 5.1	ASIA score grades of A, B, C, D, and E neurological dysfunction were reported in 10, 16, 22, 53, and 38 patients preoperatively	
Xu, 2015 [25]	Retrospective case control	Group A (intervention): 16 Group B (control): 17	Group A: 41.4 ± 4.2 Group B: 41.3 ± 3.7	Group A: 69.8 ± 4.2 Group B: 70.5 ± 5.1	Group A: 8/8 Group B: 10/7	All on lumbar region		Group A: 18.8 ± 6.3 Group B: 17.9 ± 6.2		ASIA grade: Group A: 0/1/10/5/0 Group B: 0/2/9/6/0	Group A: CVD (5), DM (3), hepatitis (7), COPD (6), other (1) Group B: CVD (2), DM (4), hepatitis (4), COPD (6), other (2)
Hassa n, 2016 [26]	Retrospective cohort	Group A (intervention): 20 Group B (control): 22	15 months (range 12–24)	Group A: 35.90 ± 10.48 Group B: 35.64 ± 12.07	Group A: 8/12 Group B: 12/15	Group A: 10/5/5 Group B: 12/6/4		Group A: 30.45 ± 15.23 Group B: 30.91 ± 16.34			
Assaghir, 2016 [27]	Retrospective cohort	Group A (intervention): 43 Group B (control): 49	Group A: 48.0 ± 7.9 (28.00–60.00) Group B: 47.5 ± 9.5 (24.00–70.00)	Group A: 49.5 ± 11.2 Group B: 47.0 ± 7.0	Group A: 16/27 Group B: 29/20			Group A: 36.6 ± 8.4 (24.0–46.0) Group B: 38.5 ± 5.9 (24.0–52.0)		Frankel scale Group A: 6/5/16/7/9 Group B: 5/9/17/7/11	
Huang, 2017 [28]	Retrospective cohort	Group A (intervention): 37 Group B (control): 149		Group A: 46.6 ± 17.3 Group B: 50.9 ± 18.0	Group A: 20/17 Group B: 91/58	T/L Group A: 18/19 Group B: 76/73		Group A: 12.2 ± 16.2 Group B: 4.6 ± 17.6	Group A: 13.2 ± 21.6 Group B: 11.7 ± 17.6	Frankel scale Group A: 0/12/2/10/24 Group B: 3/6/7/55/77	

Table 2 shows the pre-treatment, intervention, and control used for each study [9]. Most studies administered anti-TB (HREZ) chemotherapy at least 2 weeks prior to surgery apart from patients with the indication of emergency surgery. The approach to surgery was either posterior, anterior, or posterior–anterior combined. The approach was sometimes combined with debridement, instrumentation, spinal stabilization, and bone grafting when indicted. All of the included studies compared the intervention to another surgical approach [9–11, 14–28]. In total, 15 studies compared anterior to posterior approach, 4 studies compared posterior to anterior–posterior combined approach, and 1 study compared anterior to anterior–posterior combined approach [9–11, 14–28]. However, in the study conducted by Zhao et al., the posterior approach was chosen as the intervention and compared to the anterior control [16].

**Table 2:** Pre-treatment, intervention, and control of included studies

Authors	Pre-surgical treatment	Intervention (surgical management approach)	Control (surgical management approach control)
Zhang, 2012 [9]	Isoniazid (5 mg/kg), rifampicin (10 mg/kg), ethambutol (15 mg/kg) and pyrazinamide (25 mg/kg) 3–5 weeks prior to the operation	Single-stage posterior debridement, transforaminal fusion, and instrumentation	Posterior instrumentation, anterior debridement, and bone graft in a single- or two-stage procedure
Pu, 2012 [10]	Standard antituberculous chemotherapy, including oral administration of rifampin (450 mg per day), isoniazid (300 mg per day), pyrazinamide (750 mg per day) or ethambutol (1,200 mg per day), and intramuscular streptomycins (0.75 g per day). Chemotherapy lasted at least 2 weeks before the operation	Posterior approach was combined with debridement, interbody autografting, and instrumentation	Anterior approach was combined with debridement, interbody autografting, and instrumentation
Huirong, 2017 [11]		Anterior approach	Posterior approach
Yuliang, 2017 [12]	Patients received the HREZ chemotherapy regimen of isoniazid (300 mg/d), rifampicin (450 mg/d), ethambutol (750 mg/d), and pyrazinamide (1500 mg/d) preoperatively for at least 2 weeks	Anterior VATS	Posterior only
Li, 2019 [13]	All patients received quadruple standard antituberculous chemotherapy, including oral administration of isoniazid (300 mg per day), rifampin (450 mg per day), pyrazinamide (750 mg per day), and ethambutol (750 mg per day). Anti-TB chemotherapy lasted for at least 2 weeks before any surgeries	Anterior transthoracic debridement and fusion	Posterior transpedicular debridement and fusion
Sun, 2019 [14]		Anterior only approach with the ARCH plate system	Anterior + posterior
Demirel, 2019 [15]		Posterior alone	Posterior + anterior
Zhao, 2020 [16]	The patients received anti-TB treatment for at least 2 weeks before surgery. The anti-TB treatment doses consisted of 0.3 g oral quaque die (QD) of isoniazid, 0.45 g oral QD of rifampicin, 0.75 g oral QD of ethambutol, and 0.5 g ter in die (TID) of pyrazinamide, and levofloxacin 0.2 g intravenously (IV) bis in die (BID) was given during hospitalization	Anterior approach	Posterior approach
Musali, 2020 [17]		Anterolateral decompression and spinal stabilization	Posterolateral decompression by costotransversectomy and spinal stabilization
Qiu, 2022 [18]	All patients without indications of emergency surgery were routinely treated with preoperative anti-tuberculosis chemotherapy for 2–3 weeks, as well as the day of operation. The anti-tuberculosis drugs include isoniazid, rifampicin, pyrazinamide, ethambutol, and streptomycin	Anterior approach	Posterior approach
Jiang, 2022 [19]	All patients routinely received the HREZ (isoniazid: 300 mg/day, rifampicin: 450 mg/day, pyrazinamide: 750 mg, and ethambutol: 750 mg/day) chemotherapy regimen for 2–4 weeks	Posterior unilateral limited laminectomy (ULL)	Bilateral laminectomy (BL) debridement and bone grafting fusion combined with internal fixation
Wu, 2022 [20]	The patients received 2–4 weeks of HREZ standard chemotherapy regimen (including isoniazid, rifampicin, ethambutol, and pyrazinamide) before the operation	Single anterior	Single posterior
Zhang, 2012 [21]	Three weeks prior to the operation, the patients were administered an anti-tuberculosis drug with isoniazid (5–10 mg/kg/day with no more than 300 mg/day), rifampicin (5–10 mg/kg/day with no more than 300 mg/day), and ethambutol (15 mg/kg/day with no more than 500 mg/ day)	One-stage posterior debridement, TLIF and instrumentation	Posterior instrumentation, anterior debridement and bone graft in a single-stage procedure
Garg, 2012 [22]	All patients underwent four drug antituberculous chemotherapy (rifampicin, 15 mg/kg, maximum, 600 mg/day; and isoniazid, 6 mg/kg, maximum, 300 mg/day and ethambutol, 15 mg/kg, maximum 1000 mg/day and pyrazinamide, 25 mg/kg, maximum 1500 mg/day) before surgery for at least 3 weeks	Anterior debridement, decompression and instrumentation by anterior transthoracic, transpleural and/or retroperitoneal diaphragm cutting approach	Posterolateral (extracavitary) decompression and posterior instrumentation
Wang, 2014 [23]	HREZ chemotherapy (isoniazid 300 mg/day, rifampicin 450 mg/day, ethambutol 750 mg/day and pyrazinamide 750 mg/day) for 2–4 weeks prior to surgery	Anterior approach	Posterior approach
Shi, 2014 [24]		Anterior debridement and bone graft fusion with nail and screw internal fixation (nails + screws group); Posterior pedicle screw fixation (pedicle screw group)	Vertebral arch pedicle internal fixation through a posterior route (posterior arch fixation group) Posterior debridement, bone graft fusion, and vertebral arch pedicle internal fixation (arch fixation group)
Xu, 2015 [25]	All patients received standard anti-TB chemotherapy (300 mg day − 1 isoniazid, 450 mg day − 1 rifampicin, 750 mg day − 1 ethambutol, 750 mg day − 1 pyrazinamide) for an average of 2–3 weeks prior to surgery	Single- or two-stage anterior debridement, bone grafting and posterior instrumentation	Single-stage posterior debridement, decompression, interbody fusion, and instrumentation
Hassan, 2016 [26]		Anterior approach	Posterior approach
Assaghir, 2016 [27]	Chemotherapy was started 3 weeks preoperatively and continued for one year, patients were given anti-tuberculosis drugs: isoniazid 5–10 mg/kg/day (maximum 300 mg); rifampicin: 5–10 mg/kg/day (maximum 300 mg) and ethambutol 15 mg/kg/day (maximum: 500 mg) with correction of anemia and hypoproteinemia	Anterior approach	Posterior approach
Huang, 2017 [28]		Anterior approach	Posterior approach

### Clinical outcomes

Table 3 summarizes the clinical outcomes reported by the included trials. The outcomes described were duration, blood loss, follow-up correction rate, neurological function recovery, fusion time, hospital stay, and post-operative ESR and CRP. There were 17 studies that reported perioperative duration and blood loss, with 12 of them comparing anterior and posterior methods [9–11, 14, 18–21, 23–28]. With the exception of one study conducted by Assaghir et al., all of those investigations reported that the anterior approach was faster than the posterior method [27]. The same tendency was observed for blood loss, with larger amounts of blood lost when employing the posterior technique (With the exception of the study by Xu et al.) [9–11, 14, 18–21, 23–28].

**Table 3:** Outcomes of included studies

Authors	Outcomes							
	Operation duration	Blood loss	Correction rate (Kyphosis angle) on last follow-up	Recovery of neurological function	Fusion time (months)	ESR (mm/h) post-operation 3 months	CRP post-operation 3 months	Hospital Stay
Zhang, 2012 [9]	Group A: 262.1 ± 43.5 Group B: 445.6 ± 91.4	Group A: 632.5 ± 227.0 Group B: 1159.4 ± 349.4	Group A: 2.7 ± 1.9 Group B: 3.2 ± 1.4	Frankel scale post-operative: Group A: D (7); E (13) Group B: D (4); E (12)	Group A: 8.1 ± 1.8 Group B: 7.8 ± 1.7			
Pu, 2012 [10]	Group A: 390. ± 31.6 Group B: 428.6 ± 41.6	Group A: 858.1 ± 93.6 Group B: 890.9 ± 115.6	Group A: 6.1° Group B: 4.6°	All patients exhibited improvement of ASIA grade by one grade				Group A: 26.4 ± 1.2 Group B: 25.5 ± 1.4
Huirong, 2017 [11]	Group A: 392.06 ± 95.17 Group B: 378.43 ± 135.64	Group A: 748.52 ± 526.79 Group B: 802.36 ± 617.48						Group A: 27.31 ± 3.88 Group B: 30.15 ± 3.24
Yuliang, 2017 [12]	In hr Group A: 2.7 ± 0.9 Group B: 3.0 ± 0.6	Group A: 580.9 ± 163.1 Group B: 960.7 ± 295.4			Group A: 7.1 ± 1.4 Group B: 7.9 ± 1.8			Group A: 13.7 ± 2.1 Group B: 15.1 ± 2.4
Li, 2019 [13]	Group A: 158.2 ± 10.7 Group B: 183.7 ± 14.1	Group A: 517.9 ± 76.5 Group B: 714.6 ± 57.4	Angle post-operative 5-yr Group A: 9.9 ± 2.1 Group B: 10.3 ± 1.9		Group A: 6.6 ± 0.8 Group B: 8.0 ± 9.6			
Sun, 2019 [14]	Group A: 154.6 (110–220) Group B: 465.5 (185–885)	Group A: 361.1 (200–800) Group B: 814.3 (400–2700)	Lumbosacral angle Group A: 27.12 ± 2.58 Group B: 27.29 ± 1.75					Group A: 18.3 (11–33) Group B: 24.6 (17–38)
Demirel, 2019 [15]	Group A: 180.5 ± 21.5 Group B: 361.3 ± 61.3	Group A: 463 ± 52 Group B: 573 ± 57	Group A: 16.5 ± 13.6 Group B: 14.4 ± 10.2	ASIA grade post-operative: Group A: 0/0/0/2 Group B: 0/0/0/0	Group A: 10.5 ± 2.1 Group B: 9.3 ± 3.1	Group A: 7.8 ± 3.1 Group B: 8.2 ± 4.2	Group A: 6.5 ± 3.8 (1–12) Group B: 6.7 ± 4.2 (1–16)	Group A: 16.8 ± 4.8 Group B: 27.3 ± 3.4
Zhao, 2020 [16]			Correction angle Group A: 31.3 ± 16.6 Group B: 17.5 ± 4.4 Correction rate Group A: 88.6 ± 43.6% Group B: 64.9 ± 14.0%					
Musali, 2020 [17]			Correction angle Group A: 11.5 ± 3.7 Group B: 6.50 ± 2.39			Group A: 35.87 ± preoperatively to 12.7 3 ± 6.43 Group B: 37.08 ± 12.64 preoperatively to 19.83 ± 13.68 at follow-up		Group A: 12.67 ± 5.25 Group B: N/A
Qiu, 2022 [18]	Group A: 295.8 ± 115.1 Group B: 265.4 ± 65.4	Group A: 300.0 (150.0–600.0) Group B: 325.0 (140.0–850.0)	Correction angle Group A: 10.2 (3.9–16.5) Group B: 6.4 (− 0.3–16.7)	ASIA grade improvement (0/1/2 grade) Group A: 1/5/1 Group B: 1/9/0				Group A: 29.3 ± 7.3 Group B: 29.3 ± 14.0
Jiang, 2022 [19]	Group A: 192.2 ± 18.5 Group B: 160.3 ± 28.1	Group A: 611.9 ± 58.9 Group B: 469.3 ± 85.3	Correction Cobb angle Group A: 16.7 ± 5.5 Group B: 15.7 ± 6.9	ASIA grade improvement Group A: 92.9% Group B: 90.9%				Group A: 14.5 ± 2.2 Group B: 12.1 ± 2.4
Wu, 2022 [20]	Group A: 218.5 ± 15.5 Group B: 163.8 ± 13.3	Group A: 663.8 ± 82.2 Group B: 509.8 ± 72.1	Correction rate Group A: 84.2 ± 14.7% Group B: 80.2 ± 20.5%		Group A: 6.5 ± 0.8 Group B: 6.6 ± 0.7	Group A: 58.7 ± 9.4 to 13.0 ± 2.0 Group B: 57.1 ± 6.7 to 12.8 ± 2.0	Group A: 58.7 ± 13.3 to 2.8 ± 0.9 Group B: 56.4 ± 15.0 to 2.7 ± 1.0	
Zhang, 2012 [21]	Group A: 207.9 ± 30.9 Group B: 349.7 ± 38.9	Group A: 409.5 ± 107.9 Group B: 840.0 ± 168.7	Group A: 9.5 ± 1.0 Group B: 8.7 ± 0.9	Neurologic deficit was grade D in 17, all of them recovered to normal; grade B in 6, 3 recovered to grade D and 3 recovered to normal; grade C in 8, all of them went to grade E	Group A: 8.3 ± 1.7 Group B: 7.9 ± 1.9	Group A: 59.6 ± 22.7 to 9.2 ± 3.0 Group B: 61.8 ± 22.9 to 8.8 ± 2.7		
Garg, 2012 [22]			Correction rate Group A: 52.3% Group B: 72.8%	Frankel scale post-operative: Group A: 0/1/3/7/23 Group B: 0/0/2/7/27				
Wang, 2014 [23]	Group A: 231.4 ± 27.3 Group B: 160.4 ± 20.5	Group A: 1,023.8 ± 197.9 Group B: 760.7 ± 146.2	Group A: 6.4 ± 0.7 (T), 2.8 ± 0.3 (TL), 3.4 ± 0.6 (L) Group B: 11.2 ± 1.4 (T), 9.9 ± 1.5 (TL), 6.9 ± 1.7 (L)	All patients exhibited improvement of ASIA grade by one grade	Group A: fusion time ranging from 4 to 9 months (mean 7.8 months) Altogether 57 of the 60 patients obtained interbody fusion at 9.7 months (6–12 months)	Group A: 36.2 ± 5.6 to 10.2 ± 3.2 Group B: 37.1 ± 4.2 to 9.8 ± 2.3	Group A: 18.3 ± 7.5 to 3.1 ± 2.5 Group B: 21.5 ± 6.5 to 2.53 ± 1.5	Group A: 18.7 ± 3.6 Group B: 13.6 ± 3.2
Shi, 2014 [24]	Group A: 175.8 ± 48.8 Group B: 308.5 ± 76.7 Group C: 143.8 ± 43.0 Group D: 398.3 ± 90.8	Group A: 1227.1 ± 988.2 Group B: 1771.7 ± 794.7 Group C: 467.7 ± 123.3 Group D: 2833.3 ± 1083.8		ASIA grade post-operative: Group A: 2/3/6/24/36 Group B: 2/2/4/15/24 Group C: 1/0/1/5/8 Group D: 0/1/1/1/3		Group A: 15.3 ± 6.5 Group B: 12.2 ± 8.3 Group C: 17.3 ± 11.1 Group D: 18.8 ± 9.8	Group A: 4.5 ± 5.0 Group B: 7.7 ± 5.9 Group C: 9.8 ± 9.2 Group D: 7.8 ± 6.0	
Xu, 2015 [25]	Group A: 276.9 ± 23.8 Group B: 193.8 ± 22.5	Group A: 1187.5 ± 163.0 Group B: 804.7 ± 134.1	Correction angle Group A: 16.3 ± 2.0 Group B: 15.4 ± 5.0	ASIA grade Group A: 0/0/0/3/13 Group B: 0/0/0/4/13 Improvement of at least one grade in all patients	Group A: 7.8 ± 1.2 Group B: 8.4 ± 1.6	Group A: 74.6 ± 10.6 to 9.8 ± 2.9 Group B: 73.0 ± 9.8 to 9.5 ± 3.0		Group A: 18.2 ± 3.2 Group B: 13.4 ± 1.6
Hassan, 2016 [26]	Group A: 165 ± 44.07 Group B: 188.18 ± 34.87	Group A: 580.00 ± 129.17 Group B: 822.73 ± 144.53	Group A: 2.40 Group B: 1.50	Group A: neurological recovery at least one ASIA grade was 91.7% Group B: neurological recovery at least one ASIA grade was 100%				
Assaghir, 2016 [27]	Group A: 167.3 ± 16.0 Group B: 153.1 ± 23.6	Group A: 1015.2 ± 80.4 Group B: 986.6 ± 73.1	Group A: .8 ± 1.2 Group B: 1.9 ± 2.2		Group A: 19.2 ± 1.7 Group B: 18.3 ± 2.1			Group A: 6.6 ± 2.4 Group B: 7.3 ± 2.2
Huang, 2017 [28]	Group A: 212.8 ± 72.2 Group B: 224.0 ± 84.0	Group A: 723.2 ± 544.8 Group B: 748.7 ± 727.5	Post-operative cobb's angle: Group A: 2.3 ± 12.9 Group B: 0.2 ± 14.8	Frankel scale post-operative: Group A: 0/0/1/5/31 Group B: 0/1/3/17/128				Group A: 19.8 ± 8.2 Group B: 20.7 ± 32.1

When comparing the posterior and combined approaches, three investigations found that the combined strategy was faster than the posterior approach. When compared to the combined method, larger quantities of blood were lost when performing surgery using the posterior route [18–20].

In 12 of the included studies, hospitalization was reported [10–12, 14, 15, 17–19, 23, 25, 27, 28]. When comparing the anterior and posterior techniques, 5 out of 9 studies found that surgery performed with the anterior route resulted in a shorter hospital stay [10–12, 14, 15, 23, 25, 27, 28]. There was no discernible difference between the combined and posterior approaches. On the most recent follow-up, 17 research reported the corrective angle. The outcomes of the included research varied and were evenly split between anterior and posterior approaches. However, when compared to the combined strategy, three investigations found that the posterior method resulted in a higher correction rate [9, 10, 13–23, 25–28]. In addition, in the study by Zhang et al., the combination of both methods was found to be superior to the anterior approach [9]. These findings indicate that no technique was clearly superior in terms of attaining a higher correction rate.

Recovery of neurological function was largely measured using the ASIA grade or Frankel in the included studies. The findings were reported in 12 investigations [9, 10, 15, 18, 19, 21–26, 28]. In general, subjects improved their ASIA scores by one grade. When comparing anterior and posterior techniques, three research favored the posterior strategy, while four studies favored the anterior approach. In terms of neurological function, the posterior and combination approaches yielded different findings. Nine studies documented the post-operative fusion time, with mixed outcomes. Seven of the nine studies compared the anterior and posterior approaches, with four of them finding a shorter fusion time with the anterior technique. In terms of fusion time, the combination technique was reported to be faster than the anterior method [9, 12, 13, 15, 20, 21, 23, 25, 27].

Post-operative ESR and CRP were reported in seven and four studies, respectively. The decrease in ESR was comparable across the anterior and posterior approaches, with two studies favoring the posterior method significantly more [15, 17, 20, 21, 23–25].

### Complications

Nine out of twenty studies reported the complications associated with the surgical approaches (Table 4) [9, 11–13, 15, 18–20, 24]. Studies by Shi et al., Huirong et al. and Demriel et al. reported specific complications associated with the anterior and posterior approach [11, 15, 24]. Shi et al. found that wound site infection was observed in a range of patients depending on the type of fixation used, with pedicle screws showing the lowest incidence. Pneumothorax occurred in 24 patients, with primary healing observed in 127 patients, and only a small number of patients experienced local infection of the wound site or chronic sinus formation. Huirong et al. reported that incision infection, cerebrospinal fluid leakage, internal fixation loosening, and other complications occurred in both groups, with the anterior approach showing fewer complications overall. Finally, Demriel et al. found that the anterior approach experienced fewer complications overall, including a lower incidence of screw pull out, hemothorax, and pleural effusion, while posterior approach had a higher incidence of these complications, as well as non-union and rod breakage. Higher rate of complications following the posterior approach was also reported by Li et al.’s study [13]. Interestingly, in the four studies which compared the combined approach to either anterior or posterior, the anterior approach was still reported with the lowest complication rate, followed with the combined and posterior approach, respectively [9, 18–20].

**Table 4:** Complications reported of included studies

Authors	Complication
Zhang, 2012 [9]	Group A: cerebrospinal fluid leakage (1), pneumothorax (1), electrolyte imbalance (1), pneumonia Group B: cerebrospinal fluid leakage (1), pneumothorax (2), hemopneumothorax (4), electrolyte imbalance (3), pneumonia (2)
Huirong, 2017 [11]	Group A: incision infection (1), cerebrospinal fluid leakage (1), internal fixation loosening (1), others (2) Group B: incision infection (2), sinus tract (2), cerebrospinal fluid leakage (3), internal fixation loosening (2), others (4)
Yuliang, 2017 [12]	Complication rate Group A: 15.4% Group B: 13.2%
Li, 2019 [13]	Complication rate Group A: 7.7% Group B: 14.6%
Demirel, 2019 [15]	Group A: duramater injury (1), non-union (2), rod breakage (1), superficial wound infection (1) Group B: duramater injury (1), screw pull out (1), superficial wound infection (2), hemothorax (2), pleural effusion (3)
Qiu, 2022 [18]	Group A: soft tissue abscess (2), recurrence (1), pain (1), pneumothorax (3), pleural effusion (5), internal fixation displacement (1), residual kyphosis deformity (2) Group B: poor wound healing (6), soft tissue abscess (5), recurrence (4), intercostal neuralgia (1), pleural effusion (2), internal fixation displacement (1), residual kyphosis deformity (1)
Jiang, 2022 [19]	Complication rate Group A: 41.7% Group B: 16.1%
Wu, 2022 [20]	Complication rate Group A: 7.5% (3/40) Group B: 6.4% (3/47)
Shi, 2014 [24]	Wound site infection was observed in 14.1 (11/78), 14.6 (7/48), 6.2 (1/16), and 33 (2/6) patients in the nails + screws, pedicle screws, arch fixation, and posterior arch fixation groups, respectively. Pneumothorax occurred in 24 patients, with 1 case resolving spontaneously and the remaining 23 cases requiring closed thoracic drainage. Primary healing was observed in 127 patients. Local infection of the wound site was observed in 8 patients, and chronic sinus formation was observed in 6 patients, all resolving after lesion or sinus removal

### Results of meta-analysis

**Fig. 2:**
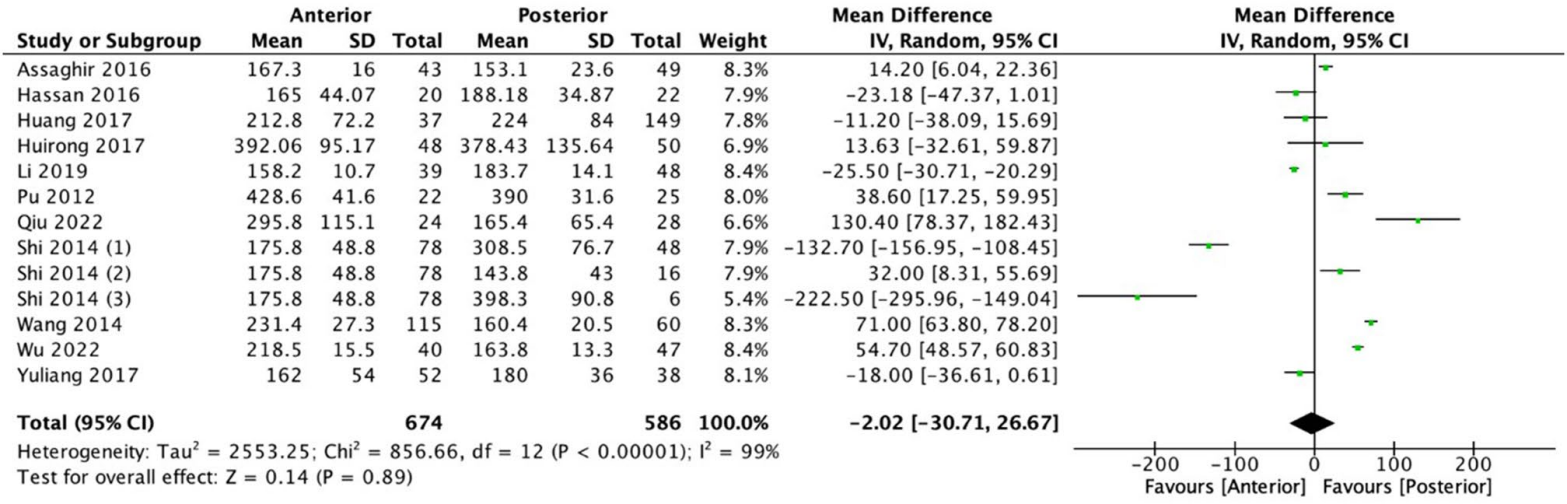
Meta-analysis and forest plot of operation duration

Meta-analysis was mostly done to compare between anterior and posterior approach (Fig. 2). There were 11 studies comparing the duration of each procedure. Six hundred seventy-four and five hundred eighty-six patients were involved for the anterior and posterior approaches, respectively. The mean difference between the two procedures is − 2.02 (− 30.71, 26.67), slightly favoring the anterior approach at the significant level (*p* < 0.00001) with high heterogeneity (*I*^2^ = 99%). Meta-analysis of three studies by Huirong et al., Pu et al., and Shi et al. demonstrated 0% heterogeneity in the sensitivity analysis [10–12, 18, 20, 23, 24, 26–28].

**Fig. 3:**
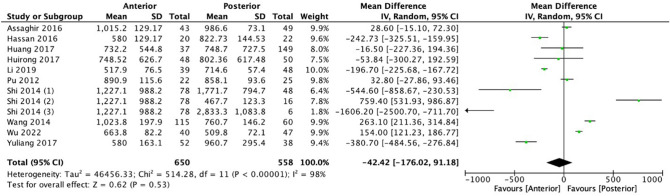
Meta-analysis and forest plot of blood loss

Ten studies were included to compare blood loss between the two procedures (Fig. 3). Six hundred fifty and five hundred fifty-eight patients were included for anterior and posterior approaches, respectively. The mean difference between the two procedures is − 4242 (− 176.02, 91.18), favoring the anterior approach at the significant level (*p* < 0.00001) with high heterogeneity (*I*^2^ = 98%). Meta-analysis of four studies conducted by Assaghir et al., Huang et al., Huirong et al., and Pu et al. showed 0% heterogeneity heterogeneity in the sensitivity analysis [10–12, 20, 23, 24, 26–28].

**Fig. 4:**
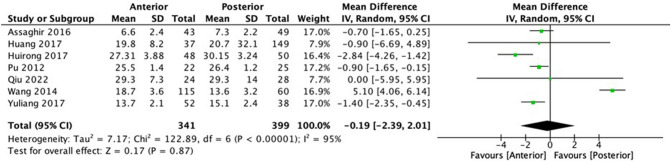
Meta-analysis and forest plot of hospital stay

Seven studies were analyzed to compare the duration of hospital stay post-operation (Fig. 4). There were 341 and 399 patients included for anterior and posterior approaches. The mean difference between the two procedures is −0.19 (−2.39, 2.01), slightly favoring the anterior approach at the significant level (p < 0.00001) with high heterogeneity (I^2^ = 95%). Only two studies conducted by Huirong et al. and Wang et al. were excluded to achieve a 0% heterogeneity in the sensitivity analysis [10–12, 18, 23, 27, 28].

**Fig. 5:**
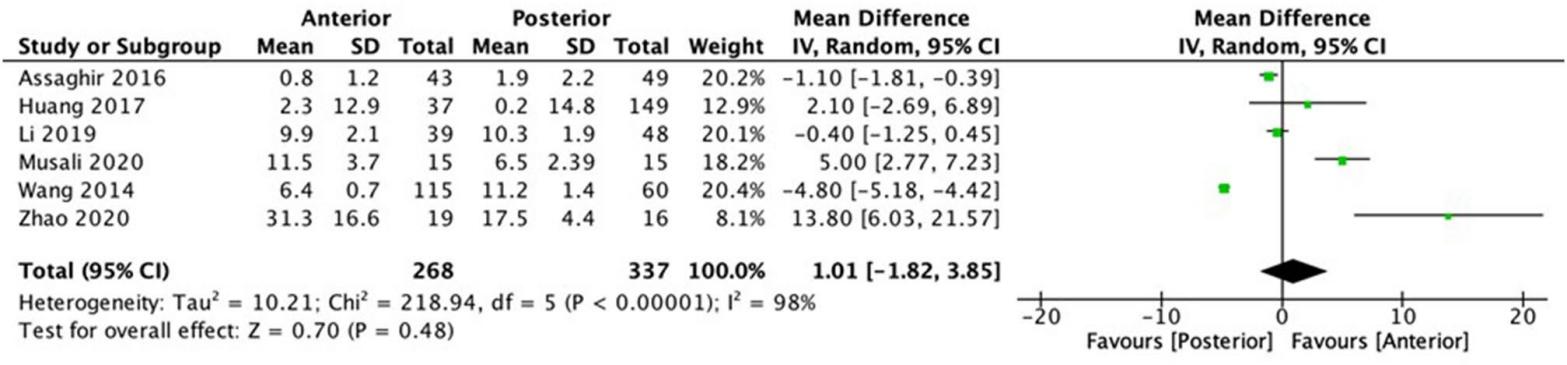
Meta-analysis and forest plot of post-operative angle correction

**Fig. 6:**
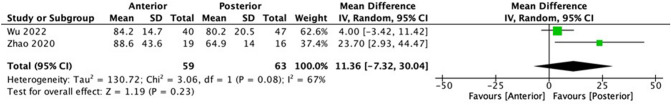
Meta-analysis and forest plot of post-operative correction rate

Correction rate as an output was divided into the angle of correction post-operation (Fig. 5**)**, and the rate correcting took place after surgery (Fig. 6**)** [13, 16, 17, 20, 23, 27, 28]. Seven studies were included to compare correction angle, with a total of three hundred forty-one and two hundred ninety-nine patients for anterior and posterior approaches, respectively. The mean difference between the two procedures is 1.01 (− 1.82, 3.85), favoring the anterior approach at the significant level (*p* < 0.00001) with high heterogeneity (*I*^2^ = 95%). Only two studies reported correction rate with a total of 59 and 63 patients included for anterior and posterior approach, respectively. Mean difference favors the posterior approach at 11.36 (− 7.32, 30.04), although non-significant (*p* < 0.08) and with moderate heterogeneity (*I*^2^ = 67%). Two studies by Assaghir et al. and Huang et al. achieved 0% heterogeneity for post-operative angle correction. Sensitivity analysis was not conducted for rate due to limited number of studies [27, 28].

### Risk of bias assessment

**Fig. 7:**
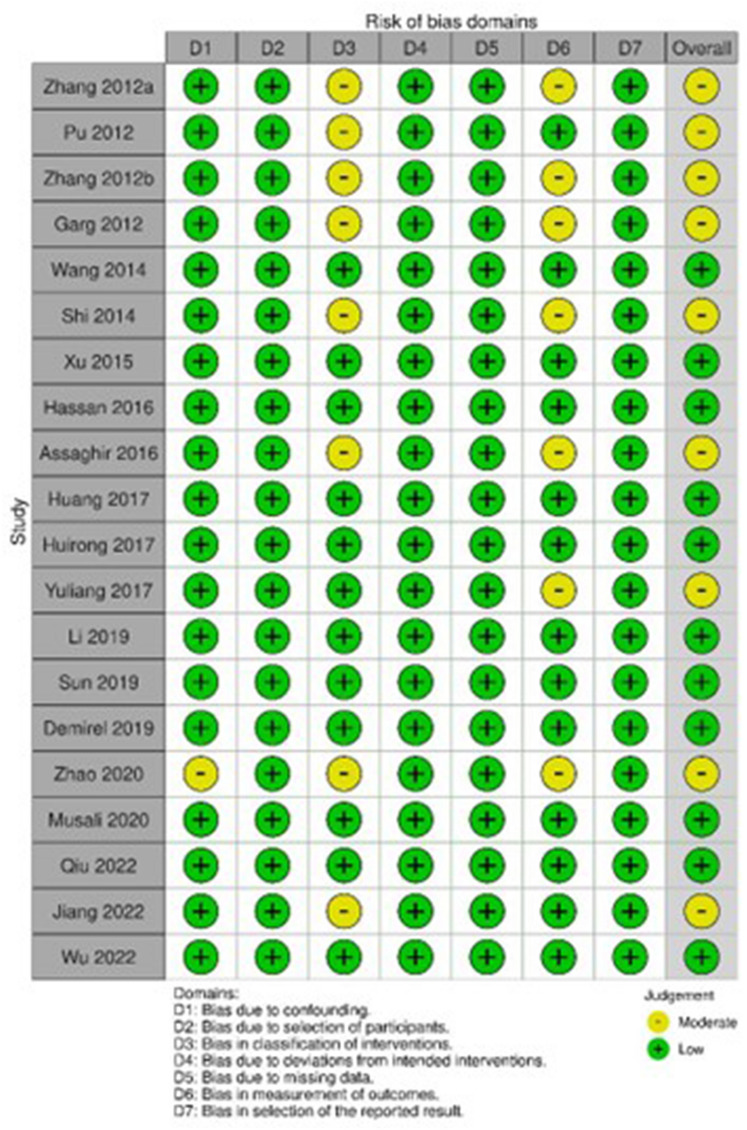
Risk of bias assessment using ROBINS

Based on ROBINS-I, 11 studies yielded low bias and 9 studies yielded moderate bias. Source of biases was mostly identified in the classification of interventions and measurement of outcomes domain (Fig. 7).

## Discussion

### Overview of spinal tuberculosis

As with its pulmonary counterpart, spinal TB is caused by a bacillus of the *Mycobacterium tuberculosis* complex. Most commonly, *M. tuberculosis, M. bovis, M. microti*, and *M. africanum* are the species found to cause TB [6]. An infection of the spine by the bacillus occurs when it spreads through the bloodstream from a primary source. The infection in the spinal marrow triggers a long-term inflammatory response that is identified by the presence of epithelioid cells, Langhans giant cells, lymphocytes, and inflammatory exudates. Together, these form a characteristic lesion called the tubercle. As the disease progresses, caseous necrosis destroys the tissue, forming a cold abscess [29]. The most common type of spinal TB is the “paradisical” variety, in which the bacilli are present in the subchondral marrow on either side of the disc. Other forms include the centrum (in which the vertebral body is destroyed), the posterior type (involving the posterior elements), and the non-osseous type (characterized by extensive abscess formation) [30].

Spinal TB is a chronic disease that starts gradually, causing pain, cold abscess, kyphotic deformity, and neurological deficit. Patients with active lesions can develop neurological deficits as a result of direct cord compression, while in late stages, it occurs due to cord stretching over an osseous ridge at the apex of the deformity. A cold abscess is a typical feature of spinal tuberculosis, occurring in at least 50% of cases. As the disease progresses, the collapse of vertebral bodies causes localized kyphotic deformity, presenting as a knuckle, gibbus, or global rounded kyphosis. Radiographs can show the earliest features of spinal tuberculosis, such as vertebral osteoporosis, narrowed joint space, and indistinct paradisical margins of vertebral bodies. With progressive destruction, vertebral collapse, kyphosis, and instability occur. CT scans can assess the extent of osseous destruction and posterior element disease, which radiographs may not show, while MRI is the preferred imaging tool for showing the extension of disease into soft tissues, the spread of tuberculous abscess, and neural compression [31, 32].

Anti-tubercular medications are usually the main approach for spinal TB. Multi-drug chemotherapy is the cornerstone of treatment. It is important because different types of bacilli exist in each colony with varying growth kinetics and metabolic characteristics. Combination drugs are also used to minimize de novo drug resistance. The most effective group of drugs against tuberculosis is the first-line drugs (isoniazid, rifampicin, ethambutol, pyrazinamide) while the second-line drugs (e.g., ciprofloxacin, levofloxacin, kanamycin) are less effective, more toxic, and expensive. Clinical effectiveness has been demonstrated through intermittent short-course chemotherapy and directly observed treatment (DOTS). Surgical approach is constrained only for spinal TB cases meeting specific indications and to prevent and/or treat complications [31].

### Current surgical strategies

After confirming that the patient adequately fulfills the indicative conditions requiring operative treatment (i.e., lack of clinical response to chemotherapy, severe and/or worsening neurological deficit, spinal instability, and severe kyphosis with late onset neurological deficits), the critical decision that spine surgeons must make is the choice of surgical approach. Currently, there are three primary approaches to surgical management of spinal tuberculosis: posterior, anterior, and combined posterior and anterior. Each of these approaches has its unique advantages and disadvantages that must be weighed against the patient’s clinical presentation and individual circumstances [32].

The posterior approach is the most commonly employed surgical strategy for spinal tuberculosis. This approach offers several advantages, including a familiar surgical corridor for most spine surgeons and less morbidity than the anterior approach. However, the posterior approach may not provide adequate exposure for anterior disease, and decompression of the spinal cord or nerve roots may be technically challenging, though surgeons have also cited the ability to achieve adequate exposure for circumferential spinal cord decompression, better deformity control via pedicle screws, possibility of extension of instrumentation if required, and avoidance of thoracotomy-related complications as benefits of the posterior approach [33]. In addition, this approach may lead to insufficient debridement of the infection, which could lead to incomplete treatment and eventual recurrence of the disease. Even so, transpedicular decompression and posterior instrumentation have been shown to support faster recovery, prevent deformity progression, and inhibit neurological sequelae in early disease [32, 34].

The anterior approach is a viable option for managing spinal tuberculosis due to its superior visualization and direct access to the infected tissue [31]. Given that spinal tuberculosis mainly affects the anterior vertebral structures, the anterior approach has been traditionally used to manage the diseased tissues directly [35]. Nevertheless, this approach is associated with serious complications, including graft-related complications (subsidence, slippage, fracture, absorption), approach-related complications (respiratory compromise), and even mortality. The anterior approach is best suited for patients without posterior vertebral structure involvement, meaning no pan vertebral disease [32].

The combined posterior and anterior approach combines the advantages of both approaches and has been shown to provide excellent clinical outcomes. However, this approach requires significant surgical experience and carries a higher risk of morbidity than either approach alone. It is of more prominent importance especially in the setting of patients with osteoporotic bones, multiple vertebral body involvement, and severe kyphotic deformities. Posterior instrumentation with anterior decompression and fusion can be performed. Anterior debridement removes the infected foci and allows for direct neural decompression and rigid reconstruction, while posterior instrumentation enables better deformity correction and reduces stress on anteriorly placed grafts, maintaining sagittal deformity correction. Due to its associated morbidities, combined approaches are only preferred for severe destructive lesions and inherently unstable junctional pathologies [36].

Minimally invasive surgery (MIS) is a relatively new approach that can be used alone or in combination with open procedures [37, 38]. It encompasses thoracoscopic debridement, posterolateral endoscopic debridement, and MIS transforaminal interbody fusion [39]. Although MIS has shown positive outcomes, especially in less severe cases, its effectiveness in cases with significant neurological deficits and extensive bone damage is uncertain [32].

### Post-surgery clinical outcomes and comparison

The operation duration indicator was assessed in multiple studies comparing different treatment options for spinal tuberculosis. In general, anterior approaches tended to be faster than posterior approaches as supported by the meta-analysis [− 2.02 (− 30.71, 26.67), *p* < 0.0001], with a few exceptions [21, 23]. In regard to blood loss, overall, anterior approaches did not consistently result in significantly more or less blood loss compared to posterior approaches, as different studies showed conflicting results. However, in the meta-analysis, the anterior approach was favored with less blood loss compared to the posterior approach [− 42.42 (− 176.02, 91.18), *p* < 0.0001]. In some comparisons, a posterior-only approach resulted in significantly less blood loss compared to a combined anterior–posterior approach [10–12, 18, 20, 23, 24, 26–28].

When compared to posterior instrumentation, anterior debridement and bone graft in a single- or two-stage procedure resulted in a better correction rate. In contrast, posterolateral decompression and posterior instrumentation showed a higher correction rate than anterior debridement, decompression and instrumentation by anterior transthoracic, transpleural and/or retroperitoneal diaphragm cutting approach. Among anterior and posterior approaches, some studies found better correction rates in the anterior approach, while others favored the posterior approach. For instance, when compared to posterior approach, the anterior approach had better results in three studies, while the posterior approach showed better correction rate in two studies. This is again supported by the meta-analysis where the anterior approach is favored in angle correction after surgery [− 0.19 (− 2.39, 2.01), *p* < 0.0001] [13, 16, 17, 20, 23, 27, 28].

Results comparing inter-study recovery of neurological function suggest that most patients had an improvement in their neurological function, regardless of the approach used. However, some studies reported that the anterior approach resulted in better neurological recovery than the posterior approach, as measured by the ASIA grade [10, 15, 18, 19, 21–26, 28]. The studies that reported this finding include Zhang et al., Wang et al., and Hassan et al. [9, 23, 26]. Conversely, the posterior approach was reported to be superior to the anterior approach in terms of neurological recovery in one study, as measured by the Frankel scale, which is Garg 2012. However, it is worth noting that the specific combinations of surgical techniques used in each study varied, making it difficult to draw definitive conclusions about which approach is superior [10, 15, 18, 19, 21–26, 28].

The anterior approach was likewise found to result in faster fusion time compared to the posterior approach in two studies. Specifically, in one study, the anterior approach yielded an average fusion time of 5 months compared to the posterior approach [21]. In another study, the anterior approach yielded an average fusion time of 7.8 months compared to the posterior approach [9, 12, 13, 15, 20, 21, 23, 25, 27]. Other studies that compared different surgical options did not report significant differences in fusion time between the groups. Overall, the findings suggest that the anterior approach may result in faster fusion time, but further work through randomized clinical trials or national registries can be done to validate this observation.

### Complications

Based on the included studies, it appears that the anterior approach may be associated with fewer complications than the posterior approach or a combined approach. Several studies, including those by Huirong et al., Demriel et al., and Li et al., reported a higher incidence of complications following the posterior approach, including screw pull out, hemothorax, pleural effusion, non-union, and rod breakage [11, 13, 15]. Meanwhile, Shi et al. found that wound site infection and pneumothorax occurred with both anterior and posterior approaches, but with varying incidence rates depending on the type of fixation used [24]. Overall, Demriel et al. reported that the anterior approach had a lower incidence of screw pull out, hemothorax, and pleural effusion, while Huirong et al. reported fewer complications overall with the anterior approach compared to the posterior approach. In addition, studies that compared the combined approach to either anterior or posterior found that the anterior approach still had the lowest complication rate, followed by the combined and posterior approach [9, 11–13, 15, 18–20, 24]. Interestingly, a different result was reported in a similar review conducted by Bian et al. In the treatment of spine tuberculosis, the incidence of surgical complications was evaluated between three surgical techniques. The anterior route had a 5.48% complications rate, the anterior combined with posterior method had a 6.62% complication rate, and the posterior approach had a 2.96% complication rate. There were statistically significant differences between the anterior and posterior approaches, as well as the anterior plus posterior strategy and the posterior approach. The anterior and anterior combined with posterior techniques had more transthoracic problems, such as pleural effusion and pneumothorax, than the posterior route. However, there was no substantial difference in the incidence of hardware failure across the three options [40].

### Limitations

Various therapies involve varying treatment procedures and doses, which can lead to biases in the actual findings. Second, due to a lack of raw data and comparative indicators, there is no full comparison of the safety of various therapies. Furthermore, the analysis conducted was restricted to studies published in English, which may have led to a language bias and excluded relevant studies conducted in other languages. Another potential limitation of our systematic review is that a significant number of the studies were assessed as having a ‘Moderate’ overall risk of bias, thus indicating some potential constraint in the design or conduct of these studies that may have affected the validity of their findings. The studies included in this systematic review had a wide variety of follow-up periods, which may not be sufficient to assess the treatments’ long-term efficacy and safety. And, if they are, they may jeopardize the findings of research with shorter follow-up periods. To give the best data for therapeutic decisions, future research should focus on the safety assessment and direct comparisons of different combination therapies. A cost-effectiveness analysis could also be considered to support the feasibility of implementation in local settings.

## Conclusion

In conclusion, spine tuberculosis is a debilitating condition that requires prompt and effective treatment to prevent complications and improve clinical outcomes. The review found that the anterior approach was associated with faster perioperative duration, less blood loss, shorter hospitalization, and better correction rates significantly compared to the posterior approach. The results of this review could help inform clinical decision-making and guide the development of future guidelines for the management of spinal tuberculosis.

## Data Availability

Data are available by request to the corresponding authors.
